# A Retrospective Study: Evaluating the Impact of the COVID-19 Pandemic on Inflammatory Markers in Hospitalized Patients

**DOI:** 10.3390/idr16040056

**Published:** 2024-08-14

**Authors:** Elmoeiz A. Elnagi, Thekra N. Al-Maqati, Rawan M. Maawadh, Salma AlBahrani, Faisal Salem Al Khalaf, Faisal M. Alzahrani, Wael Nazzal, Maha Alanazi, Abdullah S. Abdali, Amjad Saleh Al Atawi, Lamiaa H. Al-Jamea, Ahmad Mohammad Alshehri, Adnan Awad ALshammari, Rania Saad Suliman, Ibrahim Al Bassam

**Affiliations:** 1 Department of Clinical Laboratory Sciences, Prince Sultan Military College of Health Sciences, Dhahran 34313, Saudi Arabia; elmoeiz@psmchs.edu.sa (E.A.E.);; 2 Internal Medicine Department, King Fahad Military Medical Complex, Dhahran 34313, Saudi Arabia; 3 Department of Clinical Laboratory Sciences, College of Applied Medical Sciences, Imam Abdulrahman Bin Faisal University, Dammam 34212, Saudi Arabia; fmzahrani@iau.edu.sa; 4 Department of Obstetrics and Gynecology, King Fahad Military Medical Complex, Dhahran 34313, Saudi Arabia; 5 Department of Pathology and Laboratory Medicine, King Fahad Specialist Hospital, Dammam 32253, Saudi Arabia; 6 Department of Medical Laboratory, King Fahad Military Medical Complex, Dhahran 34313, Saudi Arabia; 7 Plastic and Reconstructive Surgery Unit, King Fahd Military Medical Complex, Dhahran 34313, Saudi Arabia

**Keywords:** cytokines storm, CRP, ESR, LDH, D-dimer, ferritin, PCT

## Abstract

Background: The COVID-19 pandemic has had a significant impact globally, and understanding the relationship between inflammatory markers and disease progression is crucial for effective management. This retrospective study aimed to examine the association between various inflammatory markers, such as C-reactive protein (CRP), the erythrocyte sedimentation rate (ESR), lactate dehydrogenase (LDH), D-dimer, ferritin, and procalcitonin (PCT), and the characteristics of disease progression and outcomes in individuals affected by COVID-19. Methods: This study collected raw data from 470 patients who tested positive for SARS-CoV-2 using RT-PCR. Results: The logistic regression analysis revealed that elevated LDH levels were associated with male gender, ICU admission, low oxygen saturation (O_2_ < 93%), the need for mechanical ventilation, death, and the presence of lung infiltrates. Higher D-dimer levels were associated with older age, diabetes mellitus, cardiac disease, and low oxygen saturation. Ferritin levels were significantly associated with older age, ICU admission, low oxygen saturation, mechanical ventilation, and lung infiltrates. In contrast, CRP was only significant regarding lung infiltrates and procalcitonin levels were not significantly associated with any of the examined factors. Conclusion: This study highlights the importance of monitoring key inflammatory markers, such as LDH, D-dimer, and ferritin, as they are significantly associated with the severity of COVID-19 illness. These findings can inform clinical decision-making and guide the development of targeted interventions to improve patient outcomes.

## 1. Introduction

At the end of 2019, an outbreak of a new strain of beta-coronavirus known as Severe Acute Respiratory Syndrome-Coronavirus-2 (SARS-CoV-2), which caused Coronavirus Disease-19 (COVID-19), first was documented in Wuhan, China [1,2,3]. This outbreak was increased to a global pandemic in 221 countries, including KSA, as declared by the World Health Organization (WHO) on 11 March 2020, [3,4]. In Saudi Arabia (4 May 2023), there were 841,469 confirmed cases of COVID-19 infection with 9646 deaths having been reported [5].

The clinical manifestation of COVID-19 infection ranges from asymptomatic to severe acute respiratory distress syndrome that requires intensive care unit (ICU) mechanical ventilation [6,7,8,9,10,11,12,13,14]. The most common signs and symptoms of COVID-19 infection are similar to seasonal influenza symptoms such as cough, headache, fever, nasal blockage, loss of smell (anosmia), shortness of breath (SOB), and myalgia [15,16,17,18]. In severely affected COVID-19 patients, there is a high risk of multiple vital organ dysfunction, which deteriorates the condition of these patients and increased the risk of death [15]. 

A radiological investigation of patients’ lungs through computed tomography revealed ground-glass opacities and bilateral pneumonia [17,18,19,20,21,22,23,24]. Severely affected COVID-19 cases are characterized by more severe manifestations, such as acute lung injury/respiratory distress syndrome and acute kidney injury [25]. These variations in COVID-19 infection clinical presentation (signs and symptoms) are mainly due to the alterations in the immunological response of COVID-19 patients [26,27].

The exact cause for the increased mortality rate and the severity of COVID-19 disease in some individuals is still unknown, especially in young patients who are free from comorbidities. However, the hyper-production of pro-inflammatory cytokines in response to SARS-CoV-2 together with bacterial co-infection is considered the main reason for COVID-19 disease severity and death in adults [16,19].

Usually, SARS-CoV-2 primarily infects the respiratory system, but its pathological impact can also affect other vital systems and organs [26]. COVID-19 infection starts with the viral replication stage, which is followed by an intensive immunological response in the patients. The second stage is characterized by a hyper-inflammatory response that culminates in some patients and leads to cytokine storm syndrome (CSS) [11,12]. The viral-driven intensive inflammatory reaction together with the CSS are associated with an increase in some hematological parameters, such as cytopenia, the Erythrocyte Sediment Rate (ESR), the neutrophil-to-lymphocyte ratio (lymphopenia), ferritin, and D-diner [2,11], together with the hyper-production of inflammatory parameters such as interleukin-6 (IL-6), C-reactive protein (CRP), and procalcitonin (PCT) [2,11,15,16,20,21,22]. These pro-inflammatory cytokines are extremely elevated during this stage and correlate with poor prognosis; furthermore, they also increase the risk of patient death, have a crucial role in the pathophysiological aspect of severe COVID-19 infection, and are involved in acute respiratory distress syndrome (ARDS) development, especially in high-risk patients such as elderly people and patients with diabetes mellitus or cardiovascular diseases [6,11,13,19]. The serum elevation of these inflammatory cytokines, particularly IL-6 and CRP, resulted in trans-signaling between different types of cells (immune regulation) and the eventual activation of many immunological and inflammatory cells such as macrophage/monocyte, dendritic cells, and endothelial cells, which exaggerate the inflammatory process through increasing the small blood vessel permeability of the lung, leading to acute lung injury and ARDS [15,19,20,22]. Moreover, the hyper-production of these cytokines has an important role in the pathologic hypercoagulability status of some severe cases and the stimulation of microthrombosis development in severe COVID-19 patients [15,23].

A large group of cytokine-like type I interferons (IFN-α and IFN-β) have been generated by COVID-19 patients to protect them from the viral infection [26,28]. The quick release of type I interferons by young patients of SARS-CoV-2 inhibits virus replication and might be the reason for mild disease presentation among them. However, in elderly patients, especially those with comorbidities, the delayed release of the type I interferons will lead to hyper-replication of SARS-CoV-2, which, in turn, induces the development of cytokine storm syndrome that leads to ARDS (damage to the lung alveoli), sepsis, and multi-organ damages [26,29].

Some of these pro-inflammatory markers are non-specific and their prognostic values in COVID-19 patients have not been well understood; so, the early recognition of the cytokine storm syndrome through the determination of these pro-inflammatory cytokines might have an important role in the treatment of COVID-19 patients. Therefore, the present study aims to examine the relationship between inflammatory markers such as CRP, ESR, LDH, D-dimer, ferritin, and PCT and the characteristics of disease progression and outcomes in individuals affected by COVID-19. This analysis encompasses parameters such as disease severity, admission to the intensive care unit (ICU), and laboratory indicators.

## 2. Materials and Methods

### 2.1. The Study Design

In this retrospective study, data were extracted from medical records of patients treated at a tertiary hospital in the Kingdom of Saudi Arabia during the COVID-19 pandemic from March 2020 to December 2021, without any patient identification. The Institutional Review Board of Prince Sultan Military College of Health Sciences approved this study (IRB-2023-CLS-044). For this study, the Local Research Body of Prince Sultan Military College of Health Sciences provided an exemption where patient consent was not required because this retrospective research included the extraction of data without any patient identification. Based on medical records, we collected raw data from 470 patients who tested positive for SARS-CoV-2 using RT-PCR procedures. Then, due to a result of missing data related to demographic features, comorbidities, lab results, and people younger than 18 years of age, 280 patients were excluded from the study. Study participants included 190 hepatized SARS-CoV-2-positive patients with completed variables, including demographic variables (age, gender, and BMI), comorbidities (diabetes mellitus, cardiac diseases, respiratory diseases, and end-stage renal diseases) and COVID-19 complications and outcomes (admitted to an intensive care unit, oxygen saturation below 93%, mechanical ventilation, and lung infiltrate). The lab variables were LDH, D-dimer, ferritin, CRP, and procalcitonin. The ferritin assay is a chemiluminescent microparticle immunoassay (CMIA) used to quantitatively determine ferritin levels in human serum and plasma on an Alinity i analyzer. The lactate dehydrogenase (LDH) assay quantifies LDH activity in human serum or plasma on the Alinity c system, with the rate of absorbance increase at 340 nm being proportional to the LDH activity. The D-dimer assay is a particle-enhanced, immunoturbidimetric assay for the quantitative determination of cross-linked fibrin degradation products in human plasma, using the Sysmex CS-2500/5100 Coagulation Analyzer. The Alinity c CRP Vario assay is an immunoturbidimetric assay that measures C-reactive protein (CRP) in human serum and plasma, with two different measurement ranges available on the Alinity c analyzer. The reference ranges vary by laboratory but, in general, LDH levels are considered abnormally high if they exceed 234 U/L, D-dimer levels are considered elevated if they exceed 500 ng/mL, ferritin levels are considered elevated if they exceed 336 ng/mL, CRP levels are considered elevated if they exceed 1 mg/dL, and procalcitonin levels are considered elevated if they exceed 0.1 ng/mL, with any detected level potentially indicating abnormality since the normal range is up to 0.1 ng/mL.

### 2.2. Statistical Analysis

The raw data taken from medical records were compiled and organized using Microsoft Excel (PSMCHS, Dhahran, Saudi Arabia). Missing values were deleted before the data were exported to statistical software SPSS version 26 (PSMCHS, Dhahran, Saudi Arabia) for analysis. To understand the trends in the dataset, descriptive and inferential statistical analyses were performed. Chi-square tests were used as univariate analyses for independent categorical variables to determine the association of the inflammatory markers with demographic variables and COVID-19 outcomes, as well as other comorbidities. Binary logistic regression was used to estimate the association of odd ratios with a 95% confidence interval after being found statistically significant (*p* < 0.05) in the univariate analysis.

## 3. Results

### 3.1. Characteristics of the Study Samples

The study included 191 COVID-19-positive patients. Summary statistics of all variables in the study are shown in Table 1. Out of the total sample size, 58 patients (30%) were females, while 133 patients (60%) were males. In terms of age groups, 36 patients (19%) fell within the 20–40 years, 85 patients (45%) were between 41 and 60 years old, and 70 patients (37%) were over 60 years old. When considering BMI groups, the majority of patients in this study (53%) were classified as obese, 20% were classified as having a normal BMI, and 27% were classified as overweight. Furthermore, 86 patients (45%) had been diagnosed with diabetes mellitus, while 105 patients (55%) did not have diabetes. Moreover, 38 patients (20%) had a history of cardiac disease, while the majority, 153 patients (80%), did not have cardiac disease. In addition, 27 patients (14%) were suffering from respiratory diseases, while 164 patients (86%) did not have any respiratory diseases. In terms of mortality, 16 patients (8%) had unfortunately passed away, while 175 patients (92%) were still alive. Regarding ICU admission, 69 patients (36%) had been admitted to the ICU, while 122 patients (64%) did not require ICU admission. A total of 87 patients (46%) experienced oxygen levels below 93, while 104 patients (54%) did not. Additionally, 34 patients (18%) required ventilation, while 157 patients (82%) did not. In terms of lung infiltration, 119 patients (62%) had evidence of lung infiltration, while 72 patients (38%) did not. Among the study participants, 11 patients (6%) had end-stage renal disease (ESRD), while 180 patients (94%) did not have ESRD. When considering laboratory results, 46 patients (24%) had normal LDH levels, while 145 patients (76%) had high LDH levels. Similarly, 52 patients (27%) had normal D-dimer levels, while 139 patients (73%) had high D-dimer levels. For ferritin levels, 109 patients (57%) had normal levels, while 82 patients (43%) had high levels of ferritin. In terms of C-reactive protein (CRP) levels, 32 patients (17%) had normal levels, while 159 patients (83%) had high levels. Lastly, for procalcitonin levels, 99 patients (52%) had normal levels, while 92 patients (48%) had high levels.

### 3.2. Assessment of Association between Inflammatory Markers and Demographics, Comorbidities, and Outcomes among Hospitalized COVID-19 Patients

Table 2 demonstrates the association between various variables and their impact on inflammation markers. The variables studied include demographic variables (age, gender, and BMI), comorbidities (diabetes mellitus, cardiac diseases, respiratory diseases, and end-stage renal diseases), and COVID-19 complications and outcomes (admitted to an intensive care unit, oxygen saturation below 93%, mechanical ventilation, and lung infiltrate). There was no significant association between LDH levels and gender, age group, BMI group, the presence of diabetes mellitus, cardiac disease, respiratory diseases, and ESRD. 

The results show that there is no statistically significant association between gender and LDH range (*p* = 0.0641). Similarly, the age group was found to have no significant relationship with the LDH range (*p* = 0.8434). The same was observed for the BMI group, no significant association was found (*p* = 0.5170). In terms of the medical conditions, the chi-square test revealed that diabetes mellitus (*p* = 0.1090), cardiac disease (*p* = 0.3617), and respiratory diseases (*p* = 0.4670) were not significantly associated with the LDH range. End-stage renal disease (ESRD) did not show a significant association with the LDH range (*p* = 0.1379). However, a significant association was found between the LDH range and death (*p* = 0.0138), indicating that the occurrence of death was related to LDH levels. Additionally, ICU admission (*p* < 0.0001), O_2_ < 93% (*p* = 0.0002), ventilation (*p* = 0.0062), and infiltrate (*p* = 0.0075) were all significantly associated with the LDH range.

Moreover, the analysis revealed that there was no statistically significant association between D-dimer and gender (*p* = 0.5267). Similarly, the BMI group was found to have no significant relationship with the D-dimer range (*p* = 0.3204). The same was observed for death status, no significant association was found (*p* = 0.4292). However, the age group was significantly associated with the D-dimer level (*p* = 0.0019). Moving on to medical conditions, diabetes mellitus (*p* = 0.0154), cardiac disease (*p* = 0.0.0098), respiratory diseases (*p* = 0.0424), and O_2_ < 93% (*p* = 0.0121) were all significantly associated with the D-dimer range.

When examining the association between ferritin levels and gender (*p* = 0.0607), there was no statistical association. Similarly, the BMI group was found to have no significant relationship with ferritin levels (*p* = 0.7471). On the other hand, there was a significant association between the age group and the ferritin level (*p* = 0.0082). 

Diabetes mellitus (*p* = 0.5414), cardiac disease (*p* = 0.3245), and ESRD (*p* = 0.6503) were not significantly associated with ferritin levels. However, a significant association was found between ferritin levels and death (*p* = 0.0043), indicating that the occurrence of death was related to ferritin levels. Additionally, ICU admission (*p* = 0.0043), O_2_ < 93% (*p* = 0.0018), ventilation (*p* = 0.0013), and lung infiltrate (*p* = 0.0371) were all significantly associated with ferritin levels.

The analysis revealed that there was no statistically significant association between CRP levels and gender (*p* = 0.0712). Similarly, the age group was found to have no significant relationship with CRP levels (*p* = 0.5480). The same was observed for the BMI group, where no significant association was found (*p* = 0.9816). In addition, diabetes mellitus (*p* = 0.5354), cardiac disease (*p* = 0.4279), and ESRD (*p* = 0.6943) were not significantly associated with CRP levels. However, a significant association was found between CRP levels and lung infiltrate (*p* = 0.0484). Additionally, ICU admission (*p* = 0.8592), O_2_ < 93% (*p* = 0.0750), and ventilation (*p* = 0.2433) were all found to have no statistically significant associations.

Lastly, gender, the age group, and the BMI group were not significantly associated with procalcitonin levels. The associations between procalcitonin levels and medical conditions, such as diabetes mellitus, cardiac disease, ESRD, death, ICU admission, O_2_ < 93%, ventilation, and infiltrate, were not statistically significant. 

### 3.3. Estimated Association between Inflammatory Markers as Independent Variables and Demographics, Comorbidities, and Outcomes among Hospitalized COVID-19 Patients

Table 3 shows the results from the logistic regression analysis conducted to examine the relationships between the normal level set as the reference group when compared to higher levels of inflammation markers and various independent variables. The *p*-values obtained from the logistic regression analysis were used to determine the significance of these relationships. The analysis found that there was a marginally significant association between gender and LDH levels (*p* = 0.0663). Being male is associated with a higher likelihood (OR = 1.1913) of having elevated LDH levels compared to females, although the (95% CI = 0.957–3.822) is not statistically significant. The age group, the BMI group, diabetes mellitus, cardiac disease, respiratory diseases, and ESRD were not found to have a significant association with LDH levels, as shown by the *p*-values and the 95% CIs that include 1. However, ICU admission, O_2_ < 93%, ventilation, and lung infiltrate were all found to be significantly associated with LDH levels. The odds of being admitted to the ICU when having high levels of LDH are 6.479 times higher compared to normal levels of LDH (95% CI = 2.420–17.343). In addition, the odds of having O_2_ less than 93 are about four times higher for patients with higher LDH levels compared to normal LDH levels (95% CI = 1.882–8.828). Patients with higher levels of LDH were six times more likely to require ventilation compared to those with normal levels (95% CI = 1.432–27.106). Moreover, patients with higher levels of LDH were associated with having lung infiltrate (OR = 2.482, 95% CI = 1.262–4.882). 

The analysis shows that gender was not significantly associated with D-dimer levels, as indicated by the *p*-value of 0.5272. The odds ratio (OR) of 0.795 suggests that there is no substantial association between gender and D-dimer levels, as the 95% confidence interval (CI) includes 1. The BMI group, respiratory diseases, ICU admission, ventilation, lung infiltrate, and ESRD were also not found to have a significant association with D-dimer levels. 

On the other hand, with an OR of 1.454 for the comparison of higher D-dimer levels when compared with normal levels, the age group 41–60 has 1.454 times the odds of having high D-dimer levels compared to individuals in the 20–40 age group. However, the 95% CI = 0.652–3.243 is not statistically significant. For patients above 60 years old, they have 4.8 times higher levels of D-dimer compared to those ages 20–40 years (95% CI = 1.847–12.690). 

Diabetes mellitus, cardiac disease, and O_2_ < 93% were all found to be significantly associated with higher D-dimer levels. The ORs of 2.283, 3.884, and 2.349, respectively, indicate that these factors are associated with a higher likelihood of having elevated D-dimer levels. 

When analyzing the ferritin variable, the results reveal that gender is not significantly associated with ferritin levels, as indicated by the *p*-value of 0.0624. 

The BMI group, diabetes mellitus, cardiac disease, respiratory diseases, and ESRD were also not found to have a significant association with ferritin levels, as indicated by the *p*-values and the 95% CIs that include 1.

On the other hand, the age group, ICU admission, O_2_ < 93%, ventilation, and infiltrate were all found to be significantly associated with ferritin levels. The ORs of 3.538, 1.884, 2.388, and 2.534, respectively, indicate that these factors are associated with a higher likelihood of having elevated ferritin levels. 

When examining the relationships between CRP levels and procalcitonin levels, respectively, and various independent variables, the analysis revealed the following: For CRP levels, the analysis revealed that gender, the age group, the BMI group, diabetes mellitus, cardiac disease, respiratory diseases, ICU admission, O_2_ < 93%, ventilation, and ESRD were not found to have a significant association, as indicated by the *p*-values and the 95% CIs that include the values presented in Table 1. However, lung infiltrate was found to be significant. For procalcitonin levels, gender, the age group, the BMI group, diabetes mellitus, cardiac disease, respiratory diseases, ICU admission, O_2_ < 93%, ventilation, infiltrate, and ESRD were also not found to have a significant association, as indicated by the *p*-values and the 95% CIs that include the values presented in Table 1.

## 4. Discussion

The current retrospective study of confirmed COVID-19 patients aimed to evaluate/investigate the association between the key inflammatory markers (such as C-reactive protein—CRP, lactate dehydrogenase—LDH, D-dimer, ferritin, and procalcitonin—PCT parameters), in relation to the demographic information of the COVID-19 patients (such as gender and age); the presence of comorbidities and other medical diseases (such as diabetes mellitus, cardiac disease, pulmonary/respiratory diseases, obesity/overweight, and end-stage renal disease—ESRD); and the progression of disease severity and the medical outcomes of the COVID-19 patients (such as death, admission to the intensive care unit-ICU, the need for mechanical ventilation, the presence of the lung infiltrate, and low oxygen saturation—O_2_ < 93%).

In regard to the demographic information of the hospitalized COVID-19 patients, the present study revealed that the male gender has a higher risk of developing COVID-19 infection than the female gender, as seen in several previous studies [15,30,31,32,33,34]. Our study also showed that there is no significant gender difference in relation to the measured inflammatory marker levels, which is similar to other published studies [15,35,36]. Unlike some previous studies, which showed that the male gender often exhibits more severe clinical outcomes than females, one of the clinically important explanations is the strong immunological/inflammatory activation that resulted in a significant peak in the male inflammatory marker levels [30,37,38]. In addition, the analysis of the different age groups of this study revealed that an increase in patient age contributes to disease severity, as elder patients required more mechanical ventilation than younger patients. This finding is in line with several studies of COVID-19, which have concluded that disease severity increases with age [6,38,39,40,41,42]. Several published papers showed that the mortality rate increased in elderly patients, especially those with age-associated comorbidities such as diabetes mellitus, cardiovascular diseases, and other pulmonary diseases [35,39,40]. The present study revealed that approximately 57% of the patients had one or more age-related comorbidities, with diabetes mellitus being the most prevalent one with a frequency of 45%.

Many previous studies have confirmed that age, CRP, procalcitonin, D-dimer, neutrophil, and platelet count were used as clinical outcome and severity predictors for COVID-19 infection. Therefore, they are used as important inflammatory biomarkers to predict COVID-19 prognosis and severity, because the virus induces the host’s inflammatory and immunological responses, which have a crucial role in the pathogenesis of COVID-19 infection [21,33]. This study showed that there were significantly elevated levels of LDH, D-dimer, serum ferritin, and CRP across the three age groups of COVID-19 patients. This elevation is due to the exaggerated immunological reaction and the hyper-production of the pro-inflammatory cytokines that occur during the cytokine storm syndrome (CSS) of COVID-19 infection, which may be the major factor of disease pathogenicity and mortality, and is in line with several publications [43,44,45]. In addition, the majority of studies also concluded this elevation of inflammatory cytokines, as seen in a meta-analysis of 40 studies by Melo et al. conducted in 2021 [2]. 

Our results revealed that the elevated levels of serum LDH and ferritin were mostly associated and correlated with COVID-19 severity indicators such as the requirement for mechanical ventilation (with a highly significant *p*-value of 0.0062 and 0.0013, respectively), the presence of lung infiltrates (with a highly significant *p*-value of 0.0075 and 0.0371, respectively), blood oxygen saturation level SpO_2_ < 93% (with a highly significant *p*-value of 0.0002 and 0.0018, respectively), and the need for intensive care unit (ICU) admission (with a highly significant *p*-value of 0.0001 and 0.0043, respectively). The significant association of death as a disease severity indicator with the two mentioned inflammatory cytokines was seen only for LDH (*p*-value 0.0138). This finding is correlated with previous studies, which confirm that the increment levels of LDH and ferritin could be used as a good indicators for both disease severity and poor disease prognosis, especially during the initial days of disease onset [46,47,48].

Several papers have explored the importance of the elevation of IL-6 as a key inflammatory cytokine during COVID-19 infection to predict disease severity and clinical outcome during the first days of admission, because they have significant correlations with high IL-6 [19,23,43,44]. Then, the increased levels of IL-6 trigger the formation of the acute-phase reactants such as CRP [49,50]. Therefore, it is known that the CRP is a good indicator of acute-phase response in infection, inflammation, and tissue damage [48,49,50,51]. In our study, the elevated levels of CRP were significantly consistent with these earlier studies. However, the increased CRP levels of the present study were found to have a significant correlation with only one disease severity indicator, which was the presence of lung infiltrate (*p*-value 0.0484), while no statistically significant correlations were observed for the other indicators of disease severity, such as the requirement for mechanical ventilation (*p*-value of 0.2433), the blood oxygen saturation level SpO_2_ < 93% (*p*-value of 0.075), the need for intensive care unit (ICU) admission (*p*-value of 0.8592), and death (*p*-value of 0.7349). This insignificant correlation of CRP levels with other disease severity forms might be due to the role of this acute-phase protein during the different phases/stages of COVID-19 infection, its involvement in the host’s defense mechanism, and inflammation. Furthermore, the exact role and participation of CRP in the pathogenesis of COVID-19 infection still remain unclear. However, during inflammation, CRP sensitized the infectious agents and facilitated their clearance by the host phagocytic cells as a natural immune defense; it also has anti-inflammatory actions through the inhibition of the chemical attraction of the neutrophils [52,53]. Therefore, future research should be conducted to investigate the relationship and the contribution of CRP in COVID-19 disease development and progression.

Procalcitonin (PCT) is a specific biomarker for bacterial infections; therefore, the determination of the PCT level is used in the investigation of a variety of clinical conditions, such as sepsis and septic shock. PCT is a precursor/prohormone of calcitonin [54]. Bacterial sepsis induces systemic inflammation, which stimulates PCT production in almost all tissues released into the blood stream in response to bacterial endotoxins [55] and inflammatory cytokines, such as tumor necrosis factor-alpha (TNF-α), interleukin-1-beta (IL-1β), and interleukin-6 (IL-6) [47,56,57,58]. However, in healthy individuals, PCT synthesis is restricted to the neuroendocrine cells of the thyroid and is converted to calcitonin hormone directly [47,56,59,60,61], so serum PCT levels in healthy individuals (with no systemic inflammation) are undetectable by nearly all standard PCT assays [47,60]. In contrast, most of viral infections do not induce PCT synthesis due to the production of interferon-gamma (INF-γ), which inhibits TNF-alpha production and, hence, PCT synthesis [57,59,61,62]. This study shows no significant correlation between the elevated levels of PCT and all COVID-19 disease severity indicators. The elevated serum PCT levels of our study may be due to bacterial co-infections that occur in COVID-19 infection, as seen in several previous studies [47,63].

Previous studies have clearly investigated the association of elevated levels D-dimer with poor COVID-19 outcomes, especially death and disease severity [47]. Moreover, a recent studies, has shown the formation of micro-thrombi and pulmonary microvascular- occlusion in severe COVID-19 infection [64]. Indeed, our finding regarding elevated D-dimer levels supports the strong relationship between COVID-19 infection and the disturbance of patient hemostasis, which is reflected as a hypercoagulable state in those patients [65,66,67]. So, D-dimer is confirmed to be used as a predictor factor for severe COVID-19 illness, which aligns with previous studies’ findings [47,65,66,67].

## 5. Conclusions

This study found that male COVID-19 patients have a higher risk of infection compared to females, but there were no significant gender differences in inflammatory marker levels.

Older age was associated with increased disease severity, as evidenced by a higher need for mechanical ventilation in elderly COVID-19 patients. The mortality rate also increased with age, especially in those with age-related comorbidities like diabetes, cardiovascular diseases, and pulmonary diseases. There were elevated levels of LDH, D-dimer, serum ferritin, and CRP across the three age groups of COVID-19 patients, indicating an exaggerated immunological response and cytokine storm syndrome, which may contribute to disease pathogenicity and mortality. Elevated levels of serum lactate dehydrogenase (LDH) and ferritin were strongly associated with indicators of COVID-19 severity, such as the requirement for mechanical ventilation, the presence of lung infiltrates, low blood oxygen saturation (SpO_2_ < 93%), and the need for intensive care unit admission. Elevated LDH levels were also associated with COVID-19 mortality. The exact role of CRP in COVID-19 pathogenesis remains unclear and requires further research. Procalcitonin (PCT) levels did not show significant correlation with any of the COVID-19 severity indicators. The elevated PCT levels observed were likely due to bacterial co-infections in COVID-19 patients, rather than the viral infection itself. Elevated D-dimer levels were strongly associated with COVID-19 severity and poor outcomes, supporting the notion of a hypercoagulable state in severe COVID-19 infections leading to micro-thrombosis and vascular occlusion. In summary, the study found that elevated LDH, ferritin, and D-dimer levels were reliable biomarkers for predicting COVID-19 severity and prognosis, while the role of CRP and PCT in COVID-19 pathogenesis requires further investigation.

## Figures and Tables

**Table 1:** Summary statistics of study variables (N = 191).

Demographic Variables	N (%)
Gender	
Female	58 (30%)
Male	133 (60%)
Age Group	
20–40 years	36 (19%)
41–60 years	85 (45%)
>60 years	70 (37%)
BMI Group	
Normal	38 (20%)
Overweight	52 (27%)
Obese	101 (53%)
Comorbidities Variables	N (%)
Diabetes Mellitus	
Yes	86 (45%)
No	105 (55%)
Cardiac Disease	
Yes	38 (20%)
No	153 (80%)
Respiratory Diseases	
Yes	27 (14%)
No	164 (86%)
ESRD	
Yes	11 (6%)
No	180 (94%)
COVID-19 Outcomes	N (%)
Death	
Yes	16 (8%)
No	175 (92%)
ICU Admission	
Yes	69 (36%)
No	122 (64%)
O_2_ < 93%	
Yes	87 (46%)
No	104 (54%)
Ventilation	
Yes	34 (18%)
No	157 (82%)
Infiltrate	
Yes	119 (62%)
No	72 (38%)
Inflammatory Markers	N (%)
LDH Range	
Normal	46 (24%)
High	145 (76%)
D-dimer	
Normal	52 (27%)
High	139 (73%)
Ferritin	
Normal	109 (57%)
High	82 (43%)
CRP	
Normal	32 (17%)
High	159 (83%)
Procalcitonin	
Normal	99 (52%)
High	92 (48%)

**Table 2:** Association between demographic characteristics, medical condition, and outcome variables with inflammatory markers.

	LDH Range		*p*-Value	D-Dimer Range		*p*-Value	Ferritin		*p*-Value	CRP		*p*-Value	Procalcitonin		*p*-Value
Variable	Normal	High		Normal	High		Normal	High		Normal	High		Normal	High	
Gender			0.0641			0.5267			0.0607			0.0712			0.7378
Female	19 (41%)	39 (27%)		14 (27%)	44 (32%)		39 (36%)	19 (23%)		14 (44%)	44 (28%)		29 (29%)	29 (32%)	
Male	27 (59%)	106 (73%)		38 (73%)	95 (68%)		70 (64%)	63 (77%)		18 (56%)	115 (72%)		70 (71%)	63 (68%)	
Age Group			0.8434			0.0019 *			0.0082 *			0.548			0.6072
20–40 years	10 (22%)	26 (18%)		15 (15%)	21 (15%)		27 (25%)	9 (11%)		8 (25%)	28 (18%)		16 (16%)	20 (22%)	
41–60 years	20 (43%)	65 (45%)		28 (54%)	57 (41%)		39 (36%)	46 (56%)		12 (38%)	73 (46%)		45 (44%)	40 (43%)	
>60 years	16 (35%)	54 (37%)		9 (17%)	61 (44%)		43 (40%)	27 (33%)		12 (38%)	58 (36%)		38 (38%)	32 (35%)	
BMI Group			0.517			0.3204			0.7471			0.9816			0.9926
Normal	11 (24%)	27 (19%)		8 (15%)	30 (22%)		21 (21%)	17 (21%)		6 (19%)	32 (20%)		20 (20%)	18 (20%)	
Overweight	14 (30%)	38 (26%)		18 (35%)	34 (62%)		32 (29%)	20 (24%)		9 (28%)	43 (27%)		27 (27%)	25 (27%)	
Obese	21 (46%)	80 (55%)		26 (50%)	75 (54%)		56 (51%)	45 (55%)		17 (53%)	84 (53%)		52 (53%)	49 (53%)	
Diabetes Mellitus			0.109			0.0154 *			0.5414			0.5354			0.4534
Yes	16 (35%)	70 (48%)		16 (31%)	70 (50%)		47 (43%)	39 (48%)		16 (50%)	70 (44%)		42 (42%)	44 (48%)	
No	30 (65%)	75 (52%)		36 (50%)	69 (50%)		62 (57%)	43 (52%)		16 (50%)	89 (56%)		57 (58%)	48 (52%)	
Cardiac Disease			0.3617			0.0098 *			0.3245			0.4279			0.5383
Yes	7 (15%)	31 (21%)		4 (8%)	34 (24%)		19 (17%)	19 (23%)		8 (25%)	30 (19%)		18 (18%)	20 (22%)	
No	39 (85%)	114 (79%)		48 (92%)	105 (76%)		90 (83%)	63 (77%)		24 (75%)	129 (81%)		81 (82%)	72 (78%)	
Respiratory Diseases			0.467			0.0424 *			0.1318			0.7831			0.6761
Yes	8 (17%)	19 (13%)		3 (6%)	24 (17%)		19 (17%)	8 (10%)		5 (16%)	22 (14%)		15 (15%)	12 (13%)	
No	38 (83%)	126 (87%)		49 (94%)	115 (83%)		90 (83%)	74 (90%)		22 (14%)	137 (86%)		84 (85%)	80 (87%)	
ESRD			0.1379			0.7302			0.6503			0.6943			0.1531
Yes	5 (11%)	6 (4%)		2 (4%)	9 (6%)		7 (6%)	4 (5%)		1 (3%)	10 (6%)		8 (8%)	3 (3%)	
No	41 (89%)	139 (96%)		50 (96%)	130 (94%)		102 (94%)	78 (95%)		31 (97%)	149 (94%)		91 (92%)	89 (97%)	
Death			0.0138 *			0.4292			0.0985			0.7349			0.0852
Yes	0 (0%)	16 (11%)		3 (6%)	13 (9%)		6 (5%)	10 (12%)		3 (9%)	13 (8%)		5 (5%)	11 (12%)	
No	46 (100%)	129 (89%)		49 (94%)	126 (91%)		103 (95%)	72 (88%)		29 (91%)	146 (92%)		94 (95%)	81 (88%)	
ICU Admission			<0.0001 *			0.5457			0.0043 *			0.8592			0.4046
Yes	5 (11%)	64 (44%)		17 (38%)	52 (37%)		30 (28%)	39 (48%)		12 (38%)	57 (36%)		33 (33%)	36 (39%)	
No	41 (89%)	81 (56%)		35 (67%)	87 (63%)		79 (72%)	43 (52%)		20 (63%)	102 (64%)		66 (67%)	56 (61%)	
O_2_ < 93%			0.0002 *			0.0121 *			0.0018 *			0.075			0.3683
Yes	10 (22%)	68 (47%)		16 (31%)	71 (51%)		39 (36%)	48 (59%)		10 (31%)	77 (48%)		42 (42%)	45 (49%)	
No	36 (78%)	77 (53%)		36 (69%)	68 (49%)		70 (64%)	34 (41%)		22 (69%)	82 (52%)		57 (58%)	47 (51%)	
Ventilation			0.0062 *			0.3376			0.0013 *			0.2433			0.3682
Yes	2 (4%)	32 (22%)		7 (13%)	27 (19%)		11 (10%)	23 (28%)		8 (25%)	26 (16%)		20 (20%)	14 (15%)	
No	44 (96%)	113 (78%)		45 (87%)	112 (81%)		98 (90%)	59 (72%)		24 (75%)	133 (84%)		79 (80%)	78 (85%)	
Lung Infiltrate			0.0075 *			0.0702			0.0371 *			0.0484 *			0.2714
Yes	21 (46%)	98 (68%)		27 (52%)	92 (66%)		61 (56%)	58 (71%)		15 (47%)	104 (65%)		58 (59%)	61 (66%)	
No	25 (54%)	47 (32%)		25 (48%)	47 (34%)		48 (44%)	24 (29%)		17 (53%)	55 (35%)		41 (41%)	31 (34%)	

* Significant differences at *p* < 0.05.

**Table 3:** Logistic regression model for the different inflammatory markers.

	LDH Range		*p*-Value	D-Dimer Range		*p*-Value	Ferritin		*p*-Value	CRP		*p*-Value	Procalcitonin		*p*-Value
Variable	OR	95% CI		OR	95% CI		OR	95% CI		OR	95% CI		OR	95% CI	
Gender			0.0663			0.5272			0.0624			0.0747			0.7378
Female	-	-		-	-		-	-		-	-		-	-	
Male	1.913	0.957–3.822		0.795	0.391–1.617		1.847	0.969–3.522		2.033	0.932–4.435		0.9	0.486–1.668	
Age Group			0.8438			0.0032 *			0.0098 *			0.552			0.609
20–40 years	-	-		-	-		-	-		-	-		-	-	
41–60 years	1.25	0.516–3.028		1.454	0.652–3.243		3.538	1.487–8.418		1.738	0.643–4.701		0.711	0.325–1.556	
>60 years	1.298	0.581–3.252		4.841	1.847–12.690		1.884	0.770–4.609		1.381	0.507–3.761		0.674	0.300–1.512	
BMI Group			0.519			0.3249			0.7476			0.9816			0.9926
Normal	-	-		-	-		-	-		-	-		-	-	
Overweight	1.106	0.436–2.806		0.504	0.192–1.325		0.993	0.469–2.102		0.896	0.289–2.773		1.029	0.445–2.377	
Obese	1.552	0.663–3.631		0.769	0.313–1.889		0.772	0.330–1.805		0.926	0.335–2.559		1.047	0.496–2.209	
Diabetes Mellitus			0.1113			0.0168 *			0.5415			0.5359			0.4536
Yes	1.75	0.879–3.483		2.283	1.161–4.489		1.196	0.673–2.128		0.787	0.368–1.682		1.244	0.703–2.202	
No	-	-		-	-		-	-		-	-		-	-	
Cardiac Disease			0.3644			0.0148 *			0.3267			0.4293			0.5387
Yes	1.515	0.618–3.715		3.884	1.305–11.560		1.429	0.700–2.914		0.698	0.285 - 1.704		1.25	0.614 - 2.547	
No	-	-		-	-		-	-		-	-		-	-	
Respiratory Diseases			0.4684			0.0537			0.1369			0.7912			0.6763
Yes	0.716	0.291–1.765		3.408	0.980–11.844		0.512	0.212–1.237		0.867	0.302–2.491		0.84	0.371–1.904	
No	-	-		-	-		-	-		-	-		-	-	
ESRD			0.0997			0.4925			0.6513			0.4924			0.1667
Yes	0.354	0.103–1.219		1.731	0.361–8.290		0.747	0.211–2.643		2.08	0.257–16.849		0.383	0.099–1.492	
No	-	-		-	-		-	-		-	-		-	-	
ICU Admission			0.0002 *			0.5461			0.0047 *			0.8592			0.405
Yes	6.479	2.420–17.343		1.231	0.627–2.413		2.388	1.306–4.368		0.931	0.425–2.043		1.286	0.712–2.323	
No	-	-		-	-		-	-		-	-		-	-	
O_2_ < 93%			0.0004 *			0.0133*			0.0020 *			0.079			0.3686
Yes	4.076	1.882–8.828		2.349	1.195–4.620		2.534	1.407–4.564		2.066	0.919–4.642		1.299	0.734–2.300	
No	-	-		-	-		-	-		-	-		-	-	
Ventilation			0.0147 *			0.3406			0.0020 *			0.2472			0.3695
Yes	6.23	1.432–27.106		1.549	0.630–3.812		3.473	1.580–7.635		0.586	0.238–1.448		0.709	0.334–1.503	
No	-	-		-	-		-	-		-	-		-	-	
Infiltrate			0.0084 *			0.0719			0.0382 *			0.0516			0.2721
Yes	2.482	1.262–4.882		1.813	0.948–3.464		1.902	1.036–3.492		2.143	0.995–4.616		1.391	0.772–2.507	
No	-	-		-	-		-	-		-	-		-	-	

* Significant differences at *p* < 0.05.

## Data Availability

Data are unavailable due to privacy or ethical restrictions.
